# The Royal Academy of Sciences, Paris

**Published:** 1844-06-29

**Authors:** 


### THE ROYAL ACADEMY OF SCIENCES, PARIS,

Have awarded a prize of six thousand francs in equal moieties to M. Stromeyer of Hanover, and M. Dieffenbach of Berlin,—To Stromeyer, for having first conceived the operation for squint, and shown its practicability on the dead body; to Dieffenbach, for having first successfully performed the operation on the living subject.

The Academy have further recompensed Messrs. Bourgery and Jacob with a sum of five thousand francs, for their Iconography of Surgical Anatomy and the Operations of Surgery.

The Academy have still further rewarded M. Thibert with a sum of four thousand francs for his imitations of morbid structures, in a kind of papier maché, (cartonpierre), coloured, which, say the Commissioners, "reproduce every variety of morbid structure with the greatest fidelity in regard to form, relief, and colour, and with the strictest attention to the last minutæ of detail.

The Academy have still further decreed a sum of three thousand francs to M. Longet for his work On Anatomy and Physiology of the Nervous System of Man and the Vertebrata, as containing "a great number of facts relative to the diseases of the nervous system, which scattered, and isolated hitherto, are here collected, collated, and discussed in a very complete manner. And to M. Valliez they have voted a sum of two thousand francs for his work on the Neuralgiæ, as calculated to advance the healing art, and to improve the diagnosis and therapeutics of an important class of diseases.

They have finally desired that honourable mention be made of the inquiries of M. Amussat in regard to the wounds of arteries; of the work of Messrs. Serrurier and Rousseau upon the Diseases of the Air-Passages of Man and certain Animals; and of the treatise of Dr. Ph. Boyer on the treatmeut of Ulcers by means of compression effected with strips of adhesive plaster.—*Ibid from Comptes Rendus.*

